# Estimation of Left and Right Ventricular Ejection Fractions from cine-MRI Using 3D-CNN

**DOI:** 10.3390/s23146580

**Published:** 2023-07-21

**Authors:** Soichiro Inomata, Takaaki Yoshimura, Minghui Tang, Shota Ichikawa, Hiroyuki Sugimori

**Affiliations:** 1 Graduate School of Health Sciences, Hokkaido University, Sapporo 060-0812, Japan; 2 Department of Health Sciences and Technology, Faculty of Health Sciences, Hokkaido University, Sapporo 060-0812, Japan; 3 Department of Medical Physics, Hokkaido University Hospital, Sapporo 060-8648, Japan; 4 Global Center for Biomedical Science and Engineering, Faculty of Medicine, Hokkaido University, Sapporo 060-8648, Japan; 5 Clinical AI Human Resources Development Program, Faculty of Medicine, Hokkaido University, Sapporo 060-8648, Japan; toumeiki@hs.hokudai.ac.jp; 6 Department of Diagnostic Imaging, Faculty of Medicine and Graduate School of Medicine, Hokkaido University, Sapporo 060-8638, Japan; 7 Department of Radiological Technology, School of Health Sciences, Faculty of Medicine, Niigata University, Niigata 951-8518, Japan; 8 Institute for Research Administration, Niigata University, Niigata 950-2181, Japan; 9 Department of Biomedical Science and Engineering, Faculty of Health Sciences, Hokkaido University, Sapporo 060-0812, Japan

**Keywords:** deep learning, 3D-CNN, cine-MRI

## Abstract

Cardiac function indices must be calculated using tracing from short-axis images in cine-MRI. A 3D-CNN (convolutional neural network) that adds time series information to images can estimate cardiac function indices without tracing using images with known values and cardiac cycles as the input. Since the short-axis image depicts the left and right ventricles, it is unclear which motion feature is captured. This study aims to estimate the indices by learning the short-axis images and the known left and right ventricular ejection fractions and to confirm the accuracy and whether each index is captured as a feature. A total of 100 patients with publicly available short-axis cine images were used. The dataset was divided into training:test = 8:2, and a regression model was built by training with the 3D-ResNet50. Accuracy was assessed using a five-fold cross-validation. The correlation coefficient, MAE (mean absolute error), and RMSE (root mean squared error) were determined as indices of accuracy evaluation. The mean correlation coefficient of the left ventricular ejection fraction was 0.80, MAE was 9.41, and RMSE was 12.26. The mean correlation coefficient of the right ventricular ejection fraction was 0.56, MAE was 11.35, and RMSE was 14.95. The correlation coefficient was considerably higher for the left ventricular ejection fraction. Regression modeling using the 3D-CNN indicated that the left ventricular ejection fraction was estimated more accurately, and left ventricular systolic function was captured as a feature.

## 1. Introduction

The World Health Organization (WHO) states that approximately 17.9 million people died from cardiovascular disease (CVD) in 2019, accounting for 32% of all deaths. Medical imaging has revolutionized modern medicine and healthcare, with imaging and computing technologies becoming increasingly important to the diagnosis and treatment of CVD. Computed tomography (CT), magnetic resonance imaging (MRI), positron emission tomography (PET), single-photon emission computed tomography (SPECT), and ultrasound (US) are widely used for physiological understanding and diagnostic purposes in cardiology. Specifically, CT and MRI are used to obtain specific information about the anatomy of the heart. Cardiac magnetic resonance (CMR) is an important imaging modality that allows noninvasive evaluation of myocardial disease and cardiac function [1]. The left ventricular ejection fraction (LVEF) and right ventricular ejection fraction (RVEF) are critical indices for assessing cardiac function in various clinical settings. These indices provide vital information for guiding treatment and predicting a prognosis in patients with heart disease. Specifically, the LVEF is considered an indicator in the diagnosis of CVD because early detection of CVD leads to improved cardiac function and reduced mortality [2]. With the development of CVD, the left ventricle (LV) experiences global and regional changes characterized by progressive dilation, hypertrophy, and distortion of cavity shape. Functional measurements such as ejection fractions are useful in analyzing structural changes in the LV. On the other hand, the RVEF is useful in treating patients with acute lower wall infarction complicated by right ventricular and pulmonary infarction [3]. The importance of the RVEF lies in its lower value in patients with acute lower wall infarction complicated by right ventricular infarction, obstructive pulmonary disease resulting in pulmonary hypertension, valvular disease, and congenital heart disease. Therefore, the RVEF is an essential diagnostic tool in heart disease. However, its reproducibility needs improvement due to differences in skill among observers and high interobserver variability [4]. Thus, it is crucial to develop a reliable method to measure the RVEF accurately.

Although echocardiography is widely used for measuring LVEFs and RVEFs, MRI is considered the gold standard due to its superior accuracy and reproducibility over echocardiography [5]. However, traditional MRI measurements of LVEFs and RVEFs require manual tracing of the lumen, which is a time-consuming and labor-intensive process. Furthermore, the interobserver variability is high due to the subjective nature of manual tracing. Therefore, automatic or semi-automatic methods for measuring LVEFs and RVEFs are needed to improve the accuracy and reproducibility of these indices.

In recent years, the availability of powerful graphics processing units (GPUs) has reduced computational costs and made it possible to use 3D deep learning to analyze three-dimensional (3D) medical images such as CT, ultrasound, and MRI scans, which provide detailed 3D images of human organs and can be used to detect infections, cancer, trauma, and vascular and organ abnormalities. Therefore, 3D convolutional neural networks (CNNs) are increasingly being studied with medical images, and while 2D-CNNs can extract spatial features from the input data, 3D-CNNs can be very effective in analyzing volumetric data in medical images by simultaneously extracting both spectral and spatial features from the input volume [6].

Since medical images contain a variety of information compared to normal images, the network architecture should be designed according to the characteristics of medical images; since most medical data, such as from CT and MRIs, exist in the form of 3D volume data, a 3D-CNN can be used to better extract data-specific correlations [7]. Deep learning techniques, specifically CNNs, have been applied to various medical image analyses [8,9,10,11]. CNNs are widely used for image classification [12,13,14] regression [15,16,17], object detection [18,19], super resolution [20,21], and semantic segmentation [22,23,24]. Recent studies have proposed automatic segmentation of the left ventricle lumen to reduce tracing time and interobserver errors in the study of cardiac function [25]. However, this method is computationally expensive due to the need for slice-by-slice segmentation.

To overcome these limitations, we propose the use of a 3D-CNN, a deep learning technique that extends CNN to 3D data. A 3D-CNN can extract 3D features that are more useful in comparison to a 2D-CNN [26]. There was a report [27] on the estimation of the Gleason score of the prostate by classification using a 3D-CNN; although, it was a categorical prediction as a classification instead of a directly predicted value. Specifically, we hypothesize that the LVEF and RVEF can be calculated from a series of heart movements ranging from contraction to dilation using a 3D-CNN. Since the images used to calculate the LVEF and RVEF are cine-MRI short-axis images that show both the left and right ventricles, we further hypothesize that a 3D-CNN can be used to predict both indices simultaneously. If this method can be applied clinically, it may not only improve the time efficiency of operations by reducing the manual or semi-automated complexity provided by certain conventional devices manufacturers and analysis workstations, but also minimize inter-observer errors in the calculation of LVEF and RVEF. 

We developed a 3D-CNN model to predict the LVEF and RVEF. The model was trained using 80 participants and tested using the remaining 20 participants. We used the mean absolute error (MAE) and the root mean square error (RMSE) to evaluate the model’s performance. Our results indicated that further improvements are needed to improve the prediction accuracy of the RVEF, whereas the LVEF could be estimated using the 3D-CNN with high accuracy.

The contributions of this paper are as follows:

- Proposed a method for simultaneously estimating the LVEF and RVEF from cine-MRI images of the heart.
- Compared to traditional methods, the LVEF and RVEF were estimated without the need for tracing.
- Showing the finding that the 3D-CNN captures left ventricle features rather than right ventricle features from the short-axis images.

## 2. Materials and Methods

### 2.1. Subjects and Images

This study utilized cine-MRI images from the automated cardiac diagnosis challenge (ACDC) dataset [28]. The ACDC dataset was created using real clinical examination results from the University Hospital of Dijon (Dijon, France). This dataset is the first and largest publicly available fully annotated MRI cardiac data in a medical imaging community setting. The data comprised short-axis section sequences of cardiac magnetic resonance images from 100 patients divided into five subgroups: 20 normal subjects (NOR), 20 patients with previous myocardial infarction (MINF), 20 patients with dilated cardiomyopathy (DCM), 20 patients with hypertrophic cardiomyopathy (HCM), and 20 patients with abnormal right ventricle. The spatial resolution ranged between 1.37 and 1.68 mm^2^/pixel. Our study used a 100-patient ACDC training dataset. 

**Figure 1:**
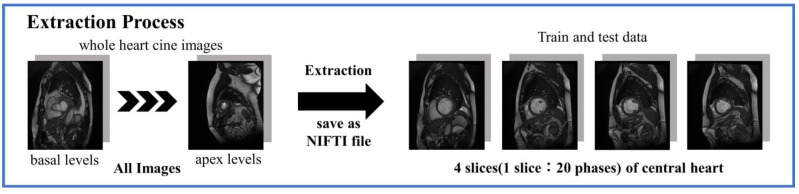
Image extraction from dataset.

These datasets contained cine-MRI images from the apex to the basal part of the heart, although the total number of slices from each subject varied. To standardize these images, the images were organized into a 3-dimensional matrix with 20 time phases per slice for a cross-section of four slices in the central region of the heart, and saved as a neuroimaging informatics technology initiative (NIFTI) file (Figure 1).

**Figure 2:**
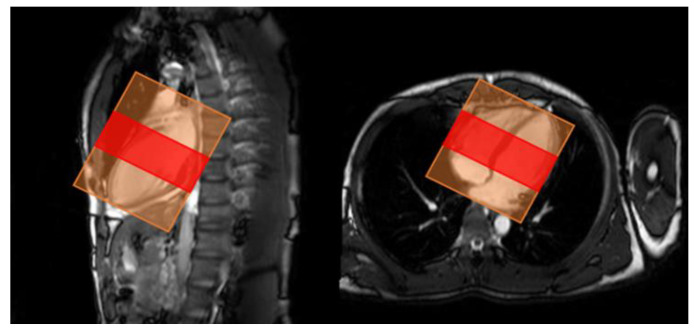
Range of slices used for analysis. Red: range used for analysis, orange: image acquisition area.

**Figure 3:**
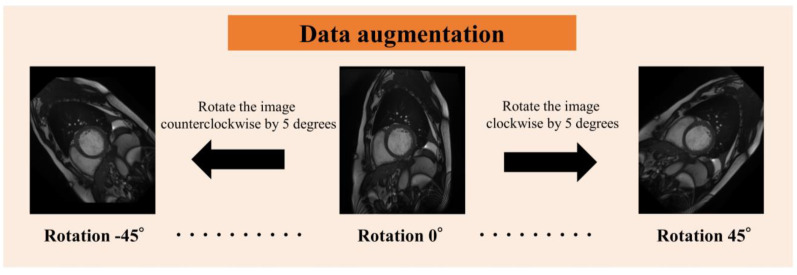
Rotation of image data for data augmentation.

Since the number of images obtained from each subject varied, and because the apex of the heart has extremely low delineation of the ventricle relative to the myocardium, and the basal part of the heart has low delineation of structures other than the left and right ventricles and myocardium, four cine-MRI slices of the mid-heart were used per subject in order to standardize the number of slices (Figure 2). We obtained 20 images per slice for a total of 80 images per subject. The images were analyzed as a single 3D image of the heart over time, from contraction to dilation. For data augmentation (Figure 3), the images were rotated in 5° increments from −45° to 45° for 19-fold expansion.

### 2.2. Analysis Method

**Figure 4:**
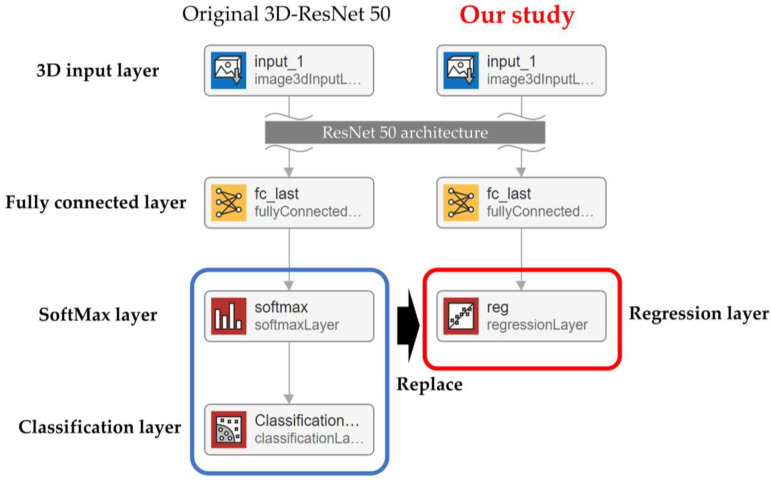
Convolutional neural network for regression in this study.

Building a regression model for estimating LVEF and RVEF from cardiac cine-MRI images required using a regression CNN based on 3D-ResNet50. This 3D-ResNet50 is a publicly available 3D-CNN for classification, in which the final layer of ResNet50, which supports 3D input, has been replaced by a regression layer (Figure 4).

Our research assessed 3D-CNN using 5-fold cross-validation (training:test = 8:2). During training, the CNN condition was the stochastic gradient descent with momentum (SGDM) optimizer, with the initial learning rate, max epochs, and mini-batch size set to 0.0001, 10, and 512, respectively. These training and analyses were performed by in-house developed software in MATLAB (2022a, The Mathworks, Natick, MA, USA). Detailed information on the computer specifications used is shown in the Table 1.

**Figure 5:**
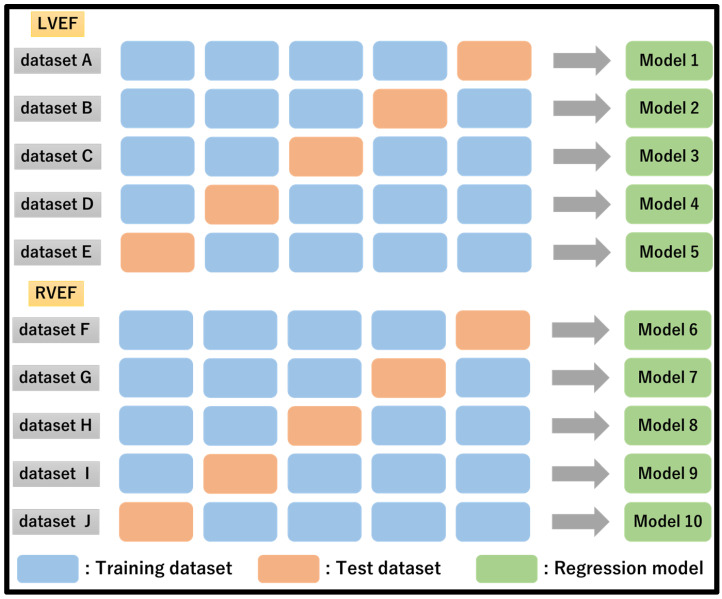
Data set creation and analysis method for LVEF and RVEF prediction.

Five subsets were created and assessed by sorting the LVEF and RVEF values in descending order to avoid bias in both LVEF and RVEF within the subset. Regression models were developed for LVEF and RVEF and tested (Figure 5).

### 2.3. Evaluation of Accuracy

**Figure 6:**
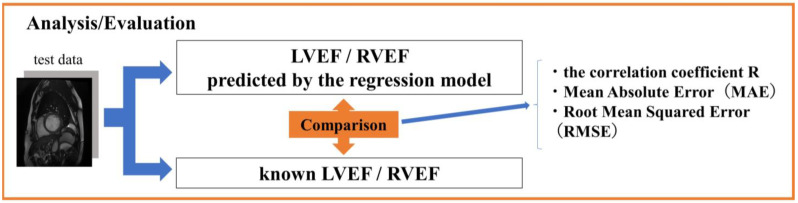
Analysis and evaluation of LVEF and RVEF predicted from regression models against known left ventricular ejection fraction LVEF and right ventricular ejection fraction RVEF.

The accuracy was measured using the correlation coefficient R, MAE, and RMSE. Correlation coefficient R was calculated using Pearson’s correlation coefficient, and a significance level of less than 0.05 was considered significant. MAE is a simple equation for calculating the evaluation measure of a regression model, called the mean absolute error between the observed and predicted values. It is used to evaluate the mean of the data set residuals. RMSE is used to represent the root mean square difference between the observed actual value and the model’s prediction. It is said to be used for absolute error representations. MAE and RMSE were calculated from the following equations:

$$MAE=\frac{\sum_{i}\left|f_{\;i}-x_{\;i}\right|}{n}$$

$$RMSE=\sqrt{\frac{\sum_{i}{\left(f_{\;i}-x_{\;i}\right)}^{2}}{n}}$$

where *f_i_* is the LVEF/RVEF predicted by the regression model, and *x_i_* is the known LVEF/RVEF. The predicted LVEF and RVEF from the regression model were evaluated by comparing them to the known LVEF and RVEF (Figure 6).

The Bland–Altman method was used to assess the errors. Bland–Altman analysis is a technique for expressing agreement between two quantitative measurements. It quantifies the agreement between two quantitative measurements by constructing limits of agreement. The resulting graph is a scatter plot XY, where the Y-axis shows the difference between two paired measurements and the X-axis shows their average. In other words, the difference between two paired measurements is plotted against the mean of the two measurements. Bland–Altman analysis assumes a 95% confidence interval with 95% of data points within ±1.96 SD (SD: standard deviation) of the mean difference.

## 3. Results

**Figure 7:**
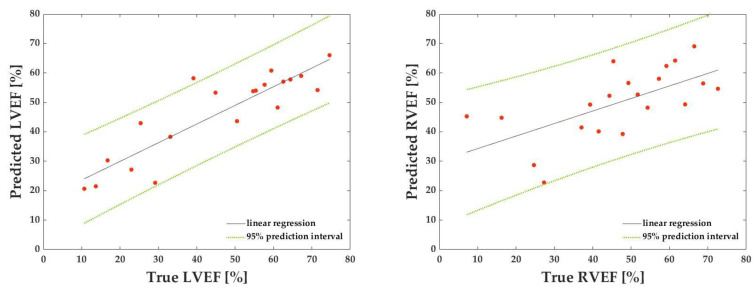
Representative linear regression lines for calculating correlation coefficients for predicted left ventricular ejection fraction (LVEF) against known LVEF (**left**) and for calculating correlation coefficients for predicted right ventricular ejection fraction (RVEF) against known RVEF (**right**). The green dashed lines represent a 95% prediction interval.

**Figure 8:**
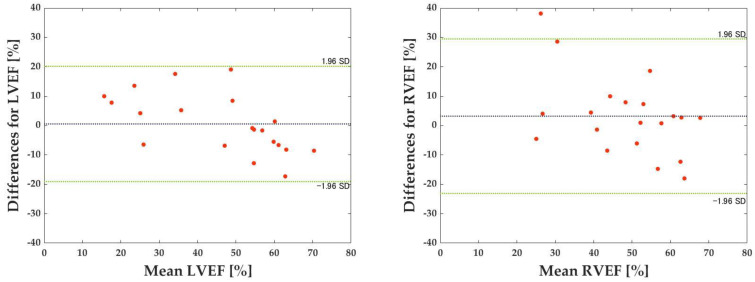
Results of Bland–Altman analysis. The x-axis represents the mean of the predicted and true values. The green dashed line represents the acceptable range of error for the mean difference ±1.96 standard deviation (SD). The black dashed lines represent the average difference between the predicted and true values.

Table 2 shows each dataset’s correlation coefficients, MAE, and RMSE for the LVEF prediction. Table 3 shows corresponding values for the RVEF prediction. The mean correlation coefficient, MAE, and RMSE for the LVEF and RVEF are also presented: the mean correlation coefficient, MAE, and RMSE for the LVEF were 0.804, 9.41, and 12.26, respectively, while the mean correlation coefficient, MAE, and RMSE for the RVEF were 0.561, 11.35, and 14.95, respectively (Table 4, Figure 7). Figure 8 depicts the results of the Bland–Altman analysis of dataset D, which was more accurate within the LVEF dataset, and dataset H, which was the best within the RVEF dataset.

**Figure 9:**
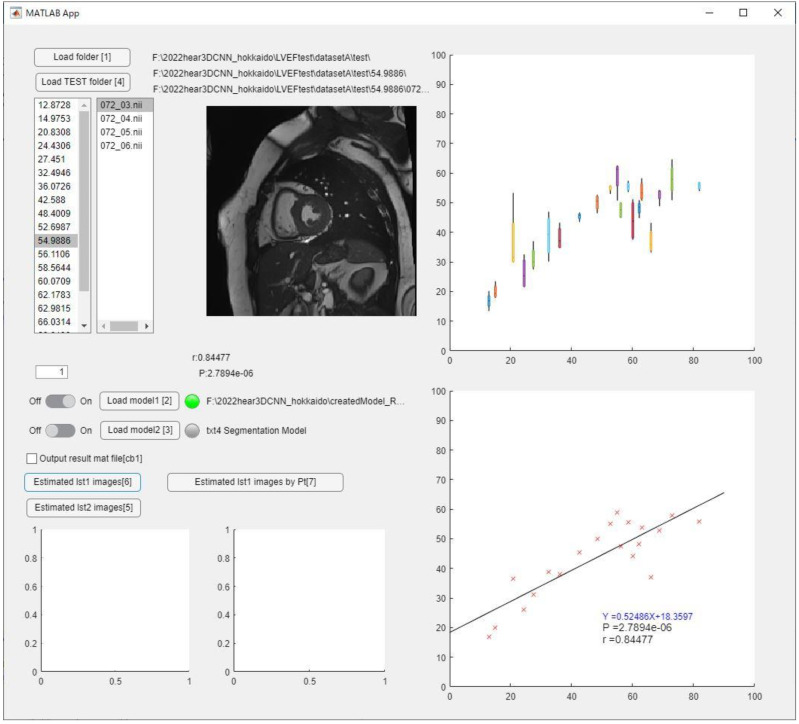
Representative analysis results on software.

Representative analysis results predicted on the software were shown in Figure 9. The LVEF or RVEF per data (20 phases) can be calculated by loading the NIFTI file as test data, and the regression line is obtained by loading all test data.

## 4. Discussion

The LVEF and RVEF are crucial indices used to evaluate cardiac function in various clinical settings. These indices provide critical information for guiding treatment and predicting prognoses in patients with heart disease. Specifically, the LVEF is an essential indicator in the diagnosis of CVD because early detection of CVD leads to improved cardiac function and reduced mortality. On the other hand, the RVEF is useful in treating patients with acute lower wall infarction complicated by right ventricular and pulmonary infarction [2]. The importance of RVEF lies in its lower value in patients with acute lower wall infarction complicated by right ventricular infarction, obstructive pulmonary disease resulting in pulmonary hypertension, valvular disease, and congenital heart disease. Therefore, RVEF is an essential diagnostic tool in heart disease. However, its reproducibility needs improvement due to differences in skill among observers and high interobserver variability. Thus, it is crucial to develop a reliable and accurate method to measure RVEF.

MRI is considered the gold standard for measuring the LVEF and RVEF due to its superior accuracy and reproducibility over echocardiography. However, traditional MRI measurements of the LVEF and RVEF require manual tracing of the lumen, which is a time-consuming and labor-intensive process. Furthermore, the interobserver variability is high due to the subjective nature of manual tracing. Therefore, automatic or semi-automatic methods for measuring the LVEF and RVEF are needed to improve the accuracy and reproducibility of these indices.

Recent advancements in deep learning techniques, specifically CNNs, have shown potential in various medical image analyses [5,6,7,8,9,10]. CNNs are widely used for image classification [11,12] and semantic segmentation [13,14]. Recent studies have proposed automatic segmentation of the left ventricle lumen to reduce tracing time and interobserver errors in the study of cardiac function [15]. However, this method is computationally expensive due to the need for slice-by-slice segmentation.

To overcome these limitations, we propose the use of a 3D-CNN, a deep learning technique that extends a CNN to 3D data. A 3D-CNN can extract 3D features that are more useful in comparison to a 2D-CNN. Specifically, we hypothesize that the LVEF and RVEF can be calculated from a series of heart movements ranging from contraction to dilation using a 3D-CNN. Since the images used to calculate the LVEF and RVEF are cine-MRI short-axis images that show both the left and right ventricles, we further hypothesize that a 3D-CNN can be used to predict both indices simultaneously.

We developed a 3D-CNN model to predict LVEF and RVEF. The model was trained using 100 participants and tested using the remaining 20 participants. We used the MAE and the RMSE to evaluate the model’s performance. Our results indicated that further improvements are needed to improve the prediction accuracy of the RVEF, whereas the LVEF could be estimated using the 3D-CNN with high accuracy.

Specifically, in this study, by training 3D (2D with time) cine-MRI short-axis images, we obtained a significant positive correlation for the LVEF, indicating that the 3D-CNN can accurately predict the movement of cardiac contraction of the left ventricle from cine-MRI short-axis images. Meanwhile, the RVEF did not demonstrate a significant positive correlation. This could be due to the fact that the short-axis images used in the analysis were set perpendicular to the line bisecting the left ventricular myocardium through the apex, possibly resulting in a significant correlation for the left ventricle. However, this finding suggests that the 3D-CNN can accurately predict the LVEF from cine-MRI short-axis images, which is clinically useful in the diagnosis and management of heart disease.

When we compared the correlation coefficients of the LVEF and RVEF, the LVEF had a greater correlation, which is consistent with previous studies. Moreover, the Bland–Altman analysis revealed that neither the LVEF nor the RVEF contained systematic errors, indicating that the 3D-CNN reads and predicts left ventricle features without the right ventricle’s influence. This is an important finding, as it suggests that the 3D-CNN model can accurately estimate the LVEF without being influenced by the right ventricle’s motion.

When comparing the accuracy of our 3D-CNN model with prior studies, we found that the MAE and RMSE were lower than those reported in previous studies [29] that predicted the LVEF without tracing and using a combination of classification and regression from 3D images of 1000 subjects, as in the current study. Specifically, our results showed an MAE of 6.84 and an RMSE of 9.74, which were better than the prior study’s results. However, since our analysis included fewer subjects than the prior study, a single dataset may not be sufficient to fully evaluate the model’s accuracy. Therefore, further studies with larger sample sizes are needed to validate the accuracy of our 3D-CNN model. In addition, the image types used in the analysis included 2-chamber and 4-chamber images as well as short-axis images, indicating that analysis of short-axis images alone is not sufficient and that adding various image types to the analysis is important for a more accurate prediction. In addition, although the subject populations were limited, external validation was not performed on the created prediction models. External validation reflects the unique image features of medical imaging devices, and there is a review paper [30] that shows that the algorithm’s performance on external data sets is diminished. Therefore, it is necessary to increase the number of subjects and to examine how the prediction results are affected by external data.

Compared to other studies that use segmentation, automatic segmentation was performed using data from 440 subjects and reported an RMSE of 5.58 and MAE of 4.08 [31]. Another study performed automatic segmentation using data from 500 subjects and reported an RMSE of 9.76 [32]. The comparison shows that segmentation is more accurate than prediction without tracing, although the number of subjects is larger than in our study. However, the regression model with the 3D-CNN can capture heart motion, as evidenced by a significant positive correlation for the LVEF in our study. While the most accurate RMSE reported in Table 2 is comparable to the RMSEs in the literature, further improvement in accuracy is needed to make our 3D-CNN model a reliable and clinically useful tool for measuring the LVEF and RVEF. The correctness of the prediction values was also compared in manual tracing and in the developed algorithm to a paper [33] published before the emergence of deep learning techniques. Since there is no baseline for assessing the level of accuracy, it is necessary to continue to improve the predictive model to achieve better robustness against manual and given supervised data.

A limitation of this research is that the LVEF and RVEF differed in the correlation coefficient, MAE, and RMSE. These differences may have been influenced by the image type used in the present study. In the present analysis, cine-MRI short-axis images were used, resulting in a left ventricular significant result. Because of the structural differences between the left and right ventricles in terms of volume, structure, and direction of contraction and dilation, the short-axis images showed a significant LVEF result, which may have caused the difference in the results. Therefore, there is potential for improving the accuracy of both the LVEF and RVEF by adding not only short-axis images but also other types of images such as two-lumen and four-lumen images to the analysis, not only to increase the number of subjects, but also to learn heart motion from various directions. In addition, since there is a possibility that other features other than the left ventricle are reflected, it is necessary to investigate in more detail in the future which structures at which level of the slice cross-section contribute the most to the prediction in the three-dimensional image input in this study. These clarifications are important for proving the validity of the results of this study and for clarifying problems and should be considered in the further investigation.

Secondly, the data augmentation will be improved. In this study, the images were rotated only by 5° from −45° to 45°. Scaling the images was not implemented in this study for the reason that enlarging the image would result in missing parts of the heart depending on the subject, and reducing the image size would make it difficult to see the heart movement. In addition, the images used in the analysis were only images of the central four slices of the heart in the cine-MRI short-axis images, where the contractile and diastolic function of the heart is clear. It is difficult to increase the number of images in the same data set because images of the base and apex of the heart have many locations other than the ventricles and myocardium, which may be underestimated in estimating the LVEF and RVEF. In the future, the number of images can be significantly increased by adding other data augmentation in addition to rotation. Additionally, the images used in the study need further consideration. The images used for training in this study were public data, and subjects were classified into five classes, including healthy subjects. However, in actual clinical practice, there are many more types of cardiac diseases, so the prediction model needs to be able to deal with uncommon cases as well. Therefore, further training on a larger number of datasets containing a variety of cases will be important in the future. Furthermore, the effects of image noise and artifacts must be considered, and Lu et al. [34] also reported a procedure that employed an algorithm to remove poor quality instances. For clinical practice, the system must be able to deal with images taken under a variety of image qualities. Therefore, since the dataset in this study only included good quality images with relatively low noise, a future challenge is to create a more generalized prediction model by training additional images that have more noise or contain artifacts such as body motion.

The varied correlation coefficient, MAE, and RMSE of the LVEF and RVEF in our study suggest that there are differences in volume, structure, and systolic and diastolic motion between the left and right ventricles. Moreover, the short-axis images used in our study may not be sufficient to capture the full motion of the right ventricle. Therefore, including other imaging modalities, such as two-chamber and four-chamber images, could increase the number of images and improve the accuracies of both the LVEF and RVEF. Additionally, since the basis of judgment for the results of this study was not visualized, revisiting these issues in the future is recommended.

## 5. Conclusions

In this study, we proposed an automatic method to estimate the LVEF and RVEF from cine-MRI images of the heart without the need for tracing compared to conventional methods. A regression 3D-CNN model that simultaneously estimates the LVEF and RVEF from cine-MRI short-axis images suggested that the LVEF can be accurately predicted using the 3D-CNN, while further improvements are needed to improve the prediction accuracy of the RVEF. Despite the potential for further improvement, our results suggest that the 3D-CNN has the potential to allow refinement in the estimation of the LVEF and RVEF, providing a faster, more accurate, and more reliable procedure for assessing cardiac function.

## Figures and Tables

**Table 1:** Software and equipment used in the study.

Environment	Contents
Software	MATLAB 2022a
OS	Windows 10
CPU	Intel core i9-10980XE 3.0 GHz
GPU	NVIDIA RTX A6000 48 GB × 2
Memory	DDR4 2933 Quad-Channel 64 GB

**Table 2:** Results of LVEF prediction.

	Correlation Coefficient R	MAE	RMSE
dataset A	0.845 (*p* < 0.05)	9.31	12.26
dataset B	0.704 (*p* < 0.05)	10.48	14.54
dataset C	0.824 (*p* < 0.05)	8.56	11.76
dataset D	0.888 (*p* < 0.05)	8.17	9.77
dataset E	0.757 (*p* < 0.05)	10.51	12.97

**Table 3:** Results of RVEF prediction.

	Correlation Coefficient R	MAE	RMSE
dataset F	0.407 (*p* = 0.08)	13.4	16.8
dataset G	0.637 (*p* < 0.05)	11.02	13.92
dataset H	0.641 (*p* < 0.05)	9.74	13.64
dataset I	0.641 (*p* < 0.05)	10.47	14.11
dataset J	0.480 (*p* < 0.05)	12.4	16.28

**Table 4:** Average of LVEF and RVEF results.

	Correlation Coefficient R	MAE	RMSE
LVEF	0.804	9.41	12.26
RVEF	0.561	11.35	14.95

## Data Availability

The created models in this study are available on request from the corresponding author. The source code of this study is available at https://github.com/MIA-laboratory/LVRVestimation (accessed on 25 April 2023).
